# A Prognostic Model Based on Six Metabolism-Related Genes in Colorectal Cancer

**DOI:** 10.1155/2020/5974350

**Published:** 2020-08-31

**Authors:** Yuan-Lin Sun, Yang Zhang, Yu-Chen Guo, Zi-Hao Yang, Yue-Chao Xu

**Affiliations:** Department of Gastrointestinal Surgery, The First Hospital, Jilin University, Changchun, 130021 Jilin Province, China

## Abstract

An increasing number of studies have shown that abnormal metabolism processes are closely correlated with the genesis and progression of colorectal cancer (CRC). In this study, we systematically explored the prognostic value of metabolism-related genes (MRGs) for CRC patients. A total of 289 differentially expressed MRGs were screened based on The Cancer Genome Atlas (TCGA) and the Molecular Signatures Database (MSigDB), and 72 differentially expressed transcription factors (TFs) were obtained from TCGA and the Cistrome Project database. The clinical samples obtained from TCGA were randomly divided at a ratio of 7 : 3 to obtain the training group (*n* = 306) and the test group (*n* = 128). After univariate and multivariate Cox regression analyses, we constructed a prognostic model based on 6 MRGs (AOC2, ENPP2, ADA, GPD1L, ACADL, and CPT2). Kaplan–Meier survival analysis of the training group, validation group, and overall samples proved that the model had statistical significance in predicting the outcomes of patients. Independent prognosis analysis suggested that this risk score might serve as an independent prognosis factor for CRC patients. Moreover, we combined the prognostic model and the clinical characteristics in a nomogram to predict the overall survival of CRC patients. Furthermore, gene set enrichment analysis (GSEA) was conducted to identify the enriched Kyoto Encyclopedia of Genes and Genomes (KEGG) pathways in the high- and low-risk groups, which might provide novel therapeutic targets for CRC patients. We discovered through the protein-protein interaction (PPI) network and TF-MRG regulatory network that 7 hub genes were retrieved from the PPI network and 4 kinds of differentially expressed TFs (NR3C1, MYH11, MAF, and CBX7) positively regulated 4 prognosis-associated MRGs (GSTM5, PTGIS, ENPP2, and P4HA3).

## 1. Introductions

Statistically, CRC is the fourth leading malignancy worldwide regarding its incidence, occupying about 10.2% of the total tumor incidence [1]. Moreover, nearly a half of patients die within 5 years after they are diagnosed [2]. Most CRC cases progress from polypoid adenomas to high-grade dysplasia, then to adenoma-adenocarcinoma [3], and this process usually takes over 10 years [3, 4]. Currently, although various treatments have been developed for CRC, the patients' prognosis still remains unsatisfactory, especially for patients with lymph node metastasis [5]. For the time being, the TNM classification system is the major pathological staging method, which can hardly accurately evaluate the prognosis of CRC. With the progress of the genome-sequencing technologies and the protein function research, an increasing number of studies about the biomarkers to predict the development and prognosis of tumor have emerged. Microsatellite Instability (MSI) status and TP53 mutation status are associated with the event-free survival after neoadjuvant chemotherapy [6]. And the high expression of PIWI-interacting RNA (piRNA) predicts poor prognosis of colorectal cancer [7]. However, the clinical application of biomarkers is still in development.

The role of metabolic disorder in the development and therapy of malignant tumors remains a research hotspot. In the mouse model, the high cholesterol levels associate with the enhanced phosphorylation of Akt, accelerating breast cancer cell growth in experiment in vitro [8]. Shu et al. believed that multiple glyceryl phosphatides, especially phosphatidylcholine and phosphatidylethanolamine, were negatively correlated with the risk of CRC [9]. To inhibit the glycolytic pathway of tumor, shikonin could suppress the activity of PKM2 [10]. Therefore, the therapy strategy targeting to the metabolism might provide novel therapeutic promise for CRC patients.

In this study, we constructed a MRG-based prognostic model to systematically evaluate the prognosis of CRC patients. Kaplan–Meier survival analysis and independent prognosis analysis demonstrated the prognostic value of the prognostic model. Next, hierarchical analysis for high- and low-risk groups in CRC patients with GSEA might provide the novel therapeutic targets for CRC patients. Moreover, the PPI network and TF-MRG network offered more reference for understanding the molecular relationship and molecular regulatory mechanisms.

## 2. Materials and Methods

### 2.1. Data Collection

The Cancer Genome Atlas (TCGA) data portal (https://portal.gdc.cancer.gov/) was used to acquire RNA sequences extracted from 482 tumor samples and 41 normal or paratumor samples and associated clinical data. The MSigDB v7.0 (c2: curated gene sets: KEGG gene sets, gene symbols) was used to obtain the MRGs. The TFs were obtained through the Cistrome Project database (http://www.cistrome.org/) [11].

### 2.2. Further Extraction of the MRG Data

The MRGs were obtained through the KEGG gene sets in MSigDB and intersected with all genes obtained in TCGA. The Wilcoxon test was utilized for differential analysis to obtain the differentially expressed MRGs according to the thresholds of false discovery rate (FDR) < 0.05 and ∣log_2_ fold change |  (∣logFC∣) > 1.

### 2.3. The Construction of the Protein-Protein Interaction (PPI) Network

In order to explore the underlying mechanisms of the interactions among the MRGs, we constructed a PPI network related to the differentially expressed MRGs with the Search Tool for the Retrieval of Interacting Genes (STRING) 11.0 (https://string-db.org). Meanwhile, Molecular Complex Detection (MCODE), the tool of the Cytoscape 3.7.2 software [12], was utilized to retrieve the hub genes of PPI network.

### 2.4. The Preliminary Validation of the Prognosis-Associated MRGs

To guarantee accuracy and objectivity, patients with missing survival time data or with a survival time of fewer than 30 days were excluded, since these patients might have died from other acute fatal diseases (heart disease and cerebral infarction), rather than CRC. Afterward, we used the “caret package” in the R software to divide patients into a training group and a validation group at a ratio of 7 : 3, and the “survival package” in the R software was employed to conduct univariate Coxregression analysis to obtain the prognosis-associated MRGs that were highly correlated with survival (*P* < 0.01). To avoid overfitting when constructing the prognostic model, the training group was subject to lasso regression analysis [13] and partial likelihood deviance to screen prognosis-associated MRGs, among which, the prognosis-associated MRGs with hazard ratio (HR) > 1 were defined as the high-risk MRGs and the prognosis-associated MRGs with hazard ratio (HR) < 1 were defined as the low-risk MRGs.

### 2.5. The Construction of the TF-MRG Network

TFs were obtained from the Cistrome Project database, and the differentially expressed TFs were extracted based on the obtained differentially expressed genes (DEGs) according to the thresholds of FDR < 0.05 and ∣logFC | >1. The prognosis-associated MRGs and differentially expressed TFs were subjected to Pearson correlation analysis (Cor > 0.4 and *P* value < 0.001) to acquire the TFs and prognosis-associated MRGs used for the construction of the TF-MRG regulatory network, which was visualized with Cytoscape 3.7.2 software.

### 2.6. The Construction of the Prognostic Model

The “survival package” and “survimer package” in R software were employed for multivariate Cox regression analysis of the training group and validation group to obtain MRGs, among which we further uncovered their functional correlations in Gene Ontology (GO) and Kyoto Encyclopedia of Genes and Genomes (KEGG) through Database for Annotation, Visualization and Integrated Discovery (DAVID) and the risk score for the construction of the prognostic model. Patients in the training group and validation group were divided into high- and low-risk groups according to the median risk score. The survival function of R software was utilized to conduct Kaplan–Meier survival analyses on the high-risk group and low-risk group in the training group, validation group, and overall samples. The risk score and survival status curves reflected the distribution of patient risk score in the high- and low-risk groups, as well as the relationship between the risk score and the survival status. Heat maps denoted the changes in the expression of various significant prognostic MRGs in the high- and low-risk groups. The clinical characteristics obtained from TCGA, including age, gender, tumor stage, pathological T stage, pathological N stage, and pathological M stage, were subjected to independent prognosis analysis combined with the risk score of the prognostic model, to verify whether the prognostic model might serve as an independent factor to predict patient prognosis. Furthermore, the “survival ROC” in R software was adopted to plot the multi-indicator ROC curves for the training group, validation group, and overall samples, and the values of the areas under the curve (AUCs) were observed to verify the feasibility and accuracy of our prognostic model and the clinical characteristics in predicting the patients' prognosis.

### 2.7. The Nomogram for the Prognostic Model and Clinical Characteristics

To more intuitively predict the patient survival time, the “rms package” in R software was used to plot the nomogram with a combination of the prognostic model and clinical characteristics (age, sex, and tumor stage). Additionally, the calibration curve and C-index were utilized to verify the consistency and integral veracity of the nomogram. The ROC curves for 1-, 3-, and 5-year patient survival were also plotted to examine the feasibility of the nomogram in predicting the patient's survival rate in chronological order.

### 2.8. Gene Set Enrichment Analysis

To judge the potential relationship of each enriched KEGG pathway in the high- and low-risk groups, the overall samples were subjected to GSEA [14]. The corresponding normalized enrichment scores (NES) in each KEGG enriched pathway were observed to judge whether this pathway was active in the high-risk group or the low-risk group. If NES > 0, the pathway was active in the high-risk group; otherwise, if NES < 0, the pathway was active in the low-risk group. The top 5 most active pathways in the high- and low-risk groups (FDR < 0.25 indicated statistical significance) were selected for further visualization analysis.

## 3. Results

### 3.1. Data Acquisition

**Figure 1:**
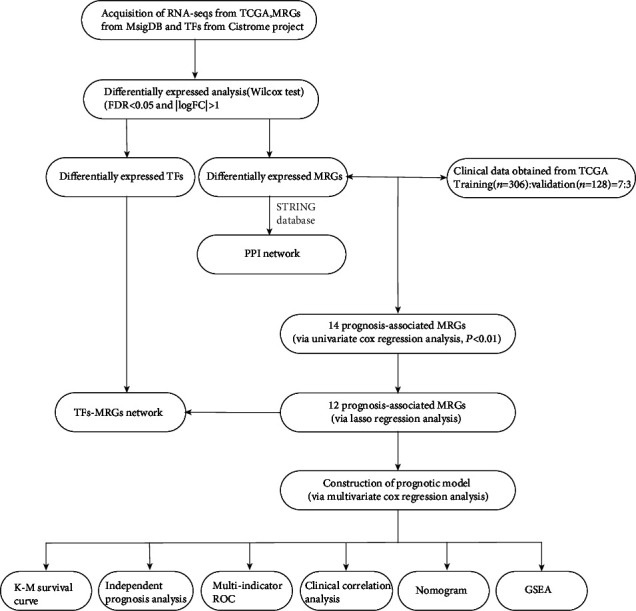
The flow diagram of the study.

**Figure 2:**
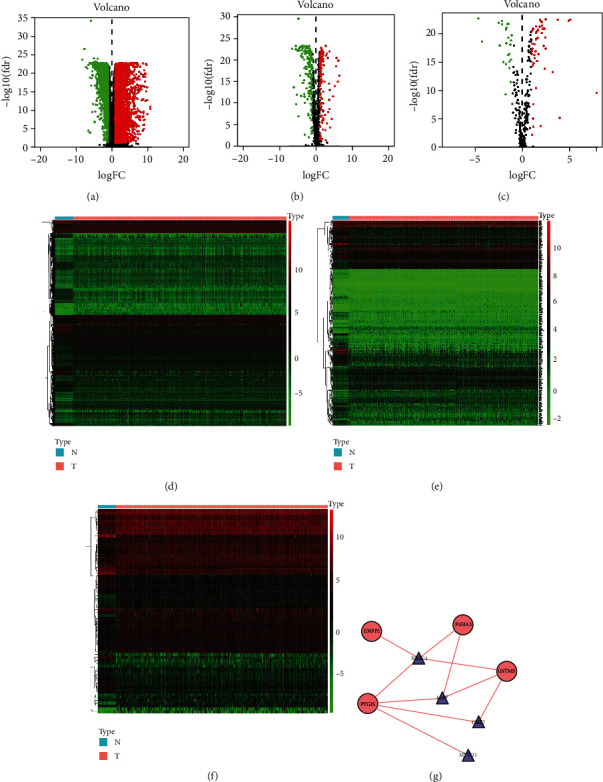
The expression of differentially expressed genes (DEGs), differentially expressed MRGs, and differentially expressed TFs. (a–f) The heat maps and volcano plots of differentially expressed genes (DEGs), differentially expressed MRGs, and differentially expressed TFs. (g) shows the TF-MRG regulatory network, in which the red line indicates a positive regulation, the triangles represent TFs, and the red ellipses represent high-risk prognosis-associated MRGs. We found that the TFs positively regulated the prognosis-associated MRGs.

The main idea of the study is shown in Figure 1. We obtained RNA sequences from 481 tumor tissues and 41 paracarcinoma or nontumor tissues from TCGA-FPKM, which were then subjected to differential analysis using the “limma package” in R software. A total of 6475 DEGs (Table S1) were obtained according to the thresholds of FDR < 0.05 and ∣logFC | >1, including 4431 upregulated and 2044 downregulated ones (Figures 2(a) and 2(d)). Besides, the genes enriched in the metabolism-related pathways were identified from the KEGG gene sets of MSigDB, which were deemed as MRGs. Altogether, 944 MRGs related to CRC were obtained after integrated analysis of the RNA sequences obtained from TCGA-FPKM, which were subjected to differential analysis by the “limma package” in R software. In line with the thresholds of FDR < 0.05 and ∣logFC | >1, 289 differentially expressed MRGs (Table S2) in total, including 163 downregulated and 126 upregulated ones, were obtained (Figures 2(b) and 2(e)). Meanwhile, 318 TFs were acquired from the Cistrome Project database, and then 72 differentially expressed TFs (Table S3) were obtained through integrated analysis of the DEGs based on the thresholds of FDR < 0.05 and ∣logFC | >1, including 47 upregulated and 25 downregulated ones (Figures 2(c) and 2(f)).

### 3.2. The PPI Network

**Figure 3:**
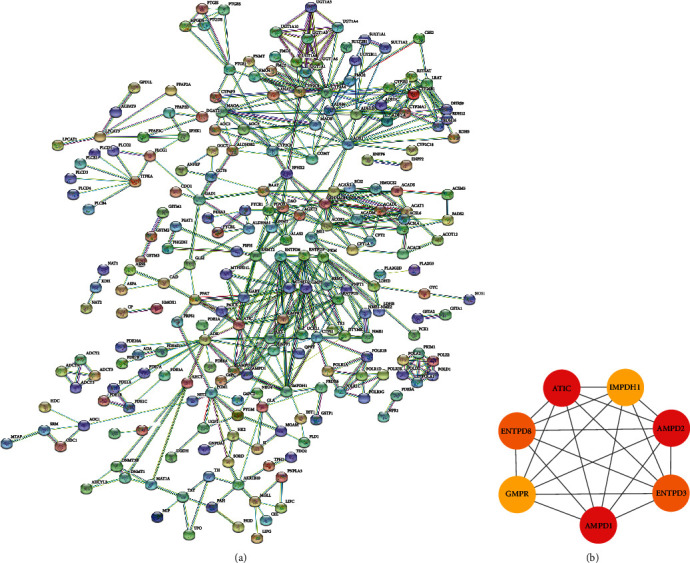
(a) The PPI network among differentially expressed MRGs. (b) The 7 hub genes of the PPI network.

We aggregately screened out 487 interaction pairs composed of 227 differentially expressed MRGs to construct the PPI network (Figure 3(a)) with a confidence of 0.900 and filtration of isolated differentially expressed MRGs. And as shown in Figure 3(b), via MCODE of Cytoscape 3.7.2, 7 hub differentially expressed MRGs were selected from the PPI network.

### 3.3. The Acquisition of Prognostic-Associated MRGs

**Figure 4:**
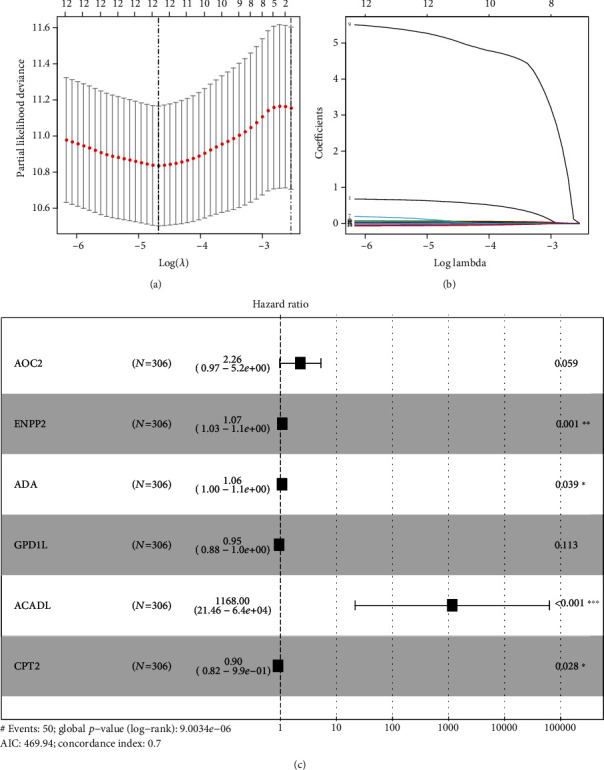
The process of constructing MRG-based prognostic model. (a, b) Lasso regression analysis and partial likelihood deviance were applied on the identification of 12 prognosis-associated MRGs in the training group. (c) The six MRGs used for the construction of the prognostic model.

Patients conforming to the screening criteria were randomly divided at a ratio of 7 : 3 into a training group (*n* = 306) and validation group (*n* = 128) (Table 1). On the basis of univariate Cox regression analysis combined with MRG expression quantities and patients' survival data, 14 MRGs significantly related to patients' survival were obtained (*P* < 0.01). Then, the 12 prognosis-associated MRGs were acquired through lasso regression analysis and partial likelihood deviance (Figures 4(a) and 4(b)). As is shown in Table 2, 9 high-risk prognosis-associated MRGs (ACADL, ENPP2, GPX3, PTGIS, ADH1B, P4HA3, ADA, AOC2, and GSTM5) (HR > 1) and 3 low-risk prognosis-associated MRGs (GPD1L, PAFAH2, and CPT2) (HR < 1) were screened out.

### 3.4. The Regulatory Network between Differentially Expressed TFs and Prognostic-Associated MRGs

We carried out Pearson correlation analysis (Cor > 0.4, *P* < 0.001) for 12 prognosis-associated MRGs and 72 DETFs and then screened 4 prognosis-associated MRGs (GSTM5, PTGIS, ENPP2, and P4HA3) and 4 differentially expressed TFs (NR3C1, MYH11, MAF, and CBX7) to construct the TF-MRG regulatory network, which was visualized using the Cytoscape 3.7.2 software for intuitive observation. Obviously, the 4 differentially expressed TFs positively regulated the 4 prognosis-associated MRGs (Figure 2(g)).

### 3.5. The Six-MRG Prognostic Model

**Figure 5:**
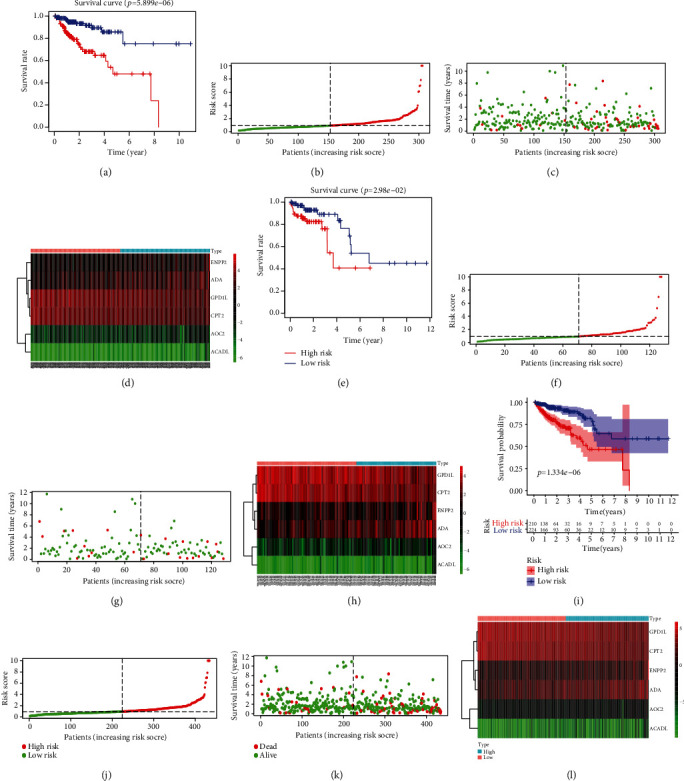
Kaplan–Meier survival analysis in training, validation, and overall samples. (a–l) Several showed the Kaplan–Meier survival curves, the risk score curves, the survival status plots, and heat maps in the training group, validation group, and overall samples.

**Figure 6:**
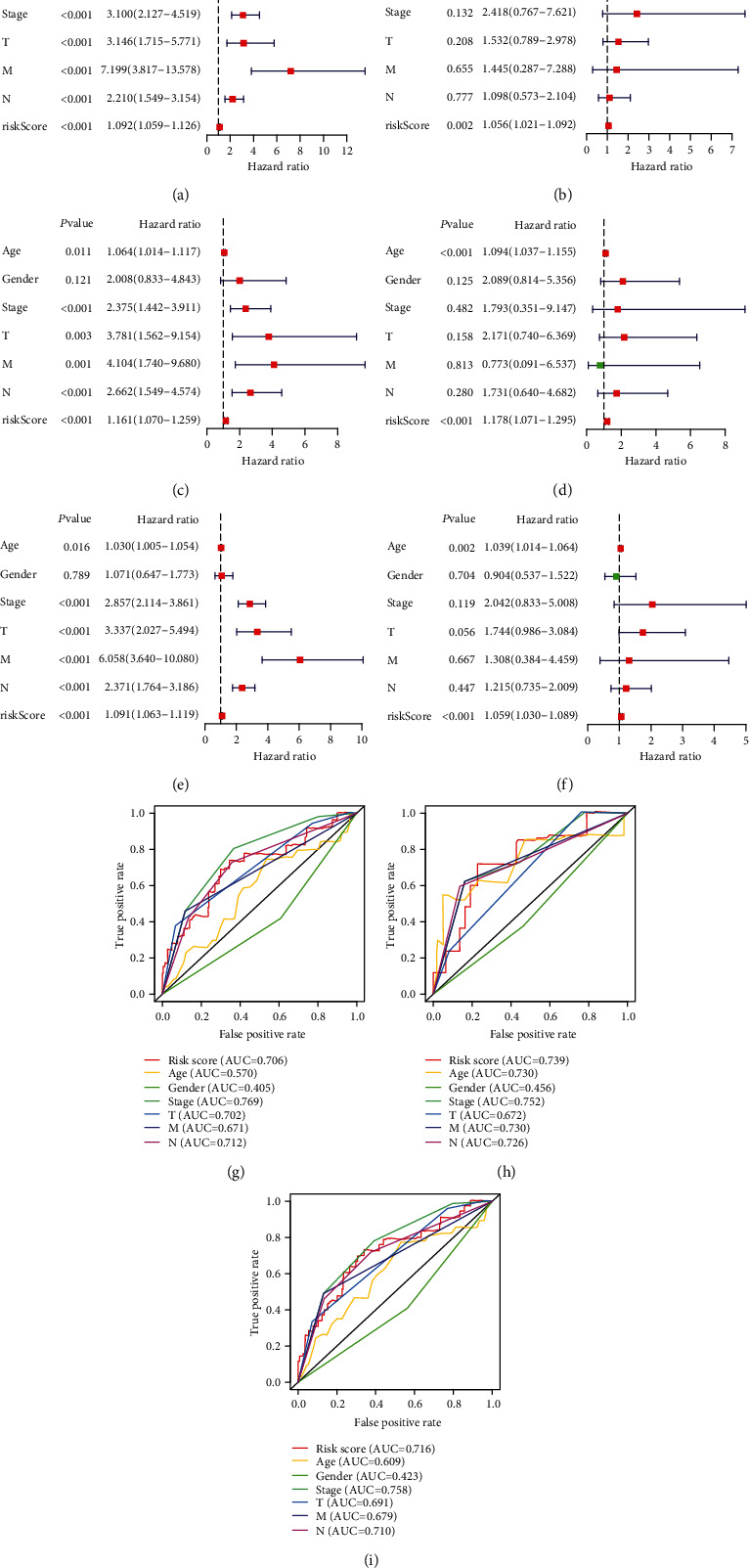
Independent prognosis analysis in training, validation, and overall samples. (a–f) Respectively showed univariate and multivariate independent prognosis analyses between clinical characteristics and risk score in the training group, validation group, and overall samples, where the red squares represent the high-risk signature and the green squares represent the low-risk signature. (g–i) The comparison of AUCs between clinical characteristics and risk score in the training group, validation group, and overall samples.

To construct the MRG-based prognostic model, we carried out multivariate Cox regression analysis on the 12 prognosis-associated MRGs. Finally, 6 MRGs (AOC2, ENPP2, ADA, ACADL, GPD1L, and CPT2), together with the corresponding coefficients, were obtained (Figure 4(c)). Eventually, the risk score was obtained, which was calculated as follows: risk score = the expression of AOC2∗0.813994 + ENPP2∗0.065392 + ADA∗0.060378 + GPD1L∗(−0.05543) + ACADL∗7.063052 + CPT2∗(−0.10342). Using the median risk score of 0.774, the patients with risk score ≥ 0.774 were classified as the high-risk group, while those with the risk score < 0.774 were classified as the low-risk group.  Table 3 shows the results of GO and KEGG functional enrichment analyses for the MRGs of the prognostic model. GPD1L, AOC2, ACADL and CPT2, ACADL and AOC2, and ACADL were, respectively, active in GO terms, oxidation-reduction process, fatty acid beta-oxidation, and electron carrier activity included, with statistical significance (*P* < 0.05). CPT2 and ACADL were all enriched in KEGG pathways including fatty acid degradation, fatty acid metabolism, and PPAR signaling pathway with statistical significance (*P* < 0.05). Figures 5(a), 5(e), and 5(i) show survival curves based on the prognostic model in the training group, validation group, and overall samples. In the training group, the 5-year survival rate for high-risk patients was about 47.9%, while that for low-risk patients, it was about 75%. In the validation group, the 5-year survival rate for high-risk patients was lower than 40.7%, and that in low-risk patients was about 60.9%. The 5-year survival rate for high-risk patients was about 46.7%, and that for low-risk patients was about 77.9%. The risk score plots (Figures 5(b), 5(f), and 5(j)) display the changes of risk score in high- and low-risk patients in training group, validation group, and overall samples, respectively. The survival status distribution graphs (Figures 5(c), 5(g), and 5(k)) show the changes of risk score and the patient survival distribution in the training group, validation group, and overall samples. Notably, the number of deaths increased and the survival time declined as the risk score increased in the training group, validation group, and overall samples. Heat maps (Figures 5(d), 5(h), and 5(l)) illustrate the variation trend of the expression of various prognosis-associated genes with the increase of the risk score. AOC2 showed the most obvious variation trend in the training group, validation group, and overall samples. Its expression quantity was positively correlated with the risk score, indicating that the expression quantity increased as the risk score increased. The expression of CPT2 and GPDL1 was negatively correlated with the risk score. To verify the influence of this prognostic model and various clinical characteristics on the patients' survival, we conducted an independent prognosis analysis. Univariate Cox regression (Table 4) and multivariate Cox regression analysis (Table 5) were performed on the clinical characteristics and risk score of the training group, validation group, and overall samples. The risk score might serve as an independent factor to predict the patient prognosis in the training group (Figures 6(a) and 6(b)), validation group (Figures 6(c) and 6(d)), and overall samples (Figures 6(e) and 6(f)) (*P* < 0.05). Additionally, the multi-indicator ROC curve was plotted, and the AUCs in the training group (Figure 6(g)), validation group (Figure 6(h)), and overall samples (Figure 6(i)) were 0.706, 0.739, and 0.716, respectively, verifying that it was more feasible and accurate to use this model in predicting the outcomes of patients than the remaining clinical indicators.

### 3.6. The Clinical Correlation Analysis

**Figure 7:**
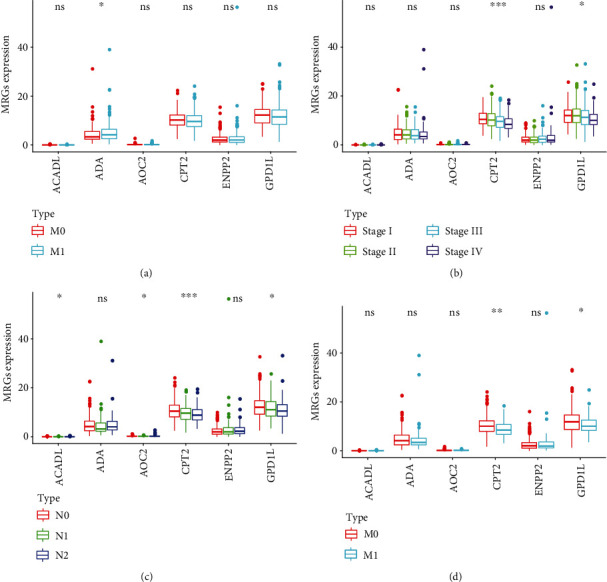
The clinical correlation analysis. (a–d) The boxplots showed the correlation between the expression level of the MRGs and the clinical characteristics, in which ^∗^*P* < 0.05, ^∗∗^*P* < 0.01, and ^∗∗∗^*P* < 0.001.

Furthermore, we carried out clinical correlation analysis on various prognosis-associated MRGs and various clinical characteristics (age, gender, tumor stage, and pathological TNM system) so as to further explore the potential molecular regulatory relationships. *P* < 0.05 was considered to denote statistical significance, while *P* < 0.01 suggested high statistical significance, and *P* < 0.001 indicated significant statistical significance. To more intuitively present their relationships, we used the “ggpubr package” of R software to plot boxplots. Clearly, as shown in Figure 7(a), the ADA expression level was correlated with age, and patients older than 60 years had slightly higher ADA expression level than those aged less than 60 years. As shown in Figure 7(b), the expression levels of CPT2 and GPDL1 were correlated with tumor stage, among which, the CPT2 expression level was significantly correlated with tumor stage. As the tumor stage advanced, the CPT2 expression level decreased accordingly, revealing a negative correlation. In tumor stage I and tumor stage II, the expression level of GPDL1 was consistent, but it decreased with the increase in tumor stage. As shown in Figure 7(c), the CPT2 expression level was considered to be significantly correlated with the pathological N stage. Patients with an advanced pathological N stage had lower expression than those with a lower pathological N stage, and the GPDL1 expression level displayed the same trend, which was related to the pathological N stage. The expression of ACADL and AOC2 showed significant correlations with pathological N stage, but their expression levels were low, with insignificant variation trends. For the pathological M stage (Figure 7(d)), the CPT2 expression level in patients with an advanced pathological M stage was lower. GPDL1 expression exhibited a similar trend, and the difference was statistically significant. The expression of the MRGs showed no obvious statistical significance with gender or pathological T stage.

### 3.7. The Four-Signature Nomogram

**Figure 8:**
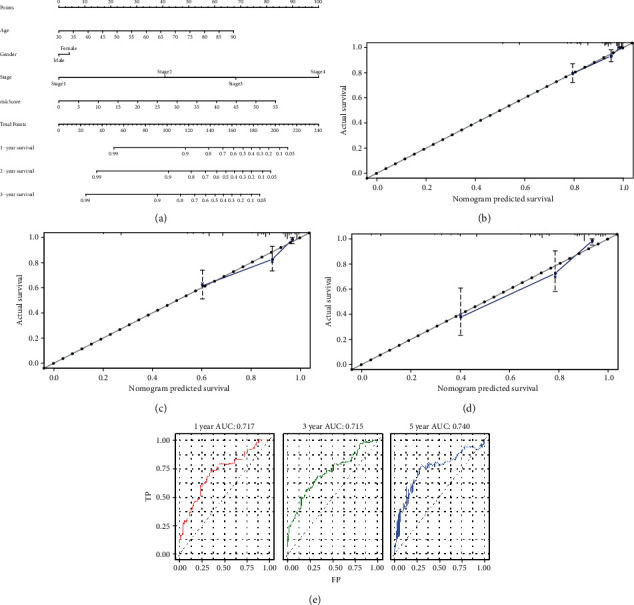
(a) The four-signature nomogram. (b–d) The 1-year, 3-year, and 5-year survival calibration curves. (e) The time-dependent ROC showed the comparison of AUCs among 1-year survival rate, 3-year survival rate, and 5-year survival rate based on the nomogram.

We integrated the prognostic model and clinical characteristics to predict patients' survival with a nomogram (Figure 8(a)). The age, gender, tumor stage, and risk score of the prognostic model were used as the elements for rating various risk factors of the patients, and the scores were added to obtain the total score, thus obtaining the corresponding predicted survival rate. Meanwhile, the 1-year, 3-year, and 5-year survival calibration curves (Figures 8(b)–8(d)) and C-index (0.806) indicated an ideal fitting and excellent accuracy of the nomogram. In the ROC curves (Figure 8(e)), the AUCs of the 1-, 3-, and 5-year survival rates were 0.717, 0.715, and 0.740, respectively, suggesting that this model relatively accurately predicted the survival rate for over 70% of the patients.

### 3.8. Gene Set Enrichment Analysis

**Figure 9:**
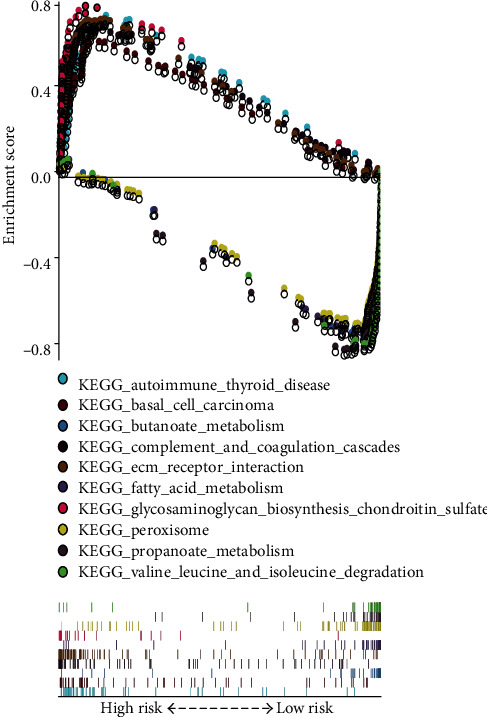
The representative ten enriched KEGG pathways in low-risk and high-risk groups conducted from GSEA.

To further explore the biological functions of the MRGs, we carried out GSEA on high- and low-risk groups, finding that 83 KEGG enriched pathways were active in the high-risk group, while 95 were active in the low-risk group. In CRC, various metabolism-related pathways were mainly enriched in the low-risk group (*n* = 38 in total, including 18 with statistical significance at FDR < 0.25, while they were rarely enriched in the high-risk group). Furthermore, 89 statistically significant KEGG enrichment pathways (FDR < 0.25) were screened, among which, 49 were active in the high-risk group, while 40 were active in the low-risk group. The top 5 pathways with the highest NES in the high- and low-risk groups (Table 6) were selected for visualization analysis (Figure 9), intuitively demonstrating that the expression of genes enriched in KEGG pathways active in the high-risk group located above the *x*-axis were apparently higher than those in the low-risk group. Genes enriched in KEGG pathways that were active in the low-risk group and located below the *x*-axis also exhibited a similar trend.

## 4. Discussion

In total, a 6-MRG- (AOC2, ENPP2, ADA, GPD1L, ACADL, and CPT2) based prognostic model was constructed based on the CRC patient clinical characteristics and expression quantity of MRGs. The exploration on AOC2 is relatively limited, and the AOC2-like enzyme activity is detected in eye tissues [15]. ENPP2 is a gene that encodes autotaxin, which has been verified to be related to the growth and metastasis of melanoma tumor and stage I nonsmall cell lung cancer (NSCLC) [16, 17]. And Zhao et al. revealed that autotaxin protein encoded by ENPP2 catalyzes the production of LPC into lysophosphatidic acid (LPA), and such lipid molecular metabolic reaction may be associated with the genesis and development of CRC [18]. Some studies indicated that ADA, participating in encoding an enzyme involved in purine metabolism, is downregulated in lymphocytes of advanced stage lung cancer [19]. Kelly et al. suggested that GPDL1 negatively regulated HIF-1*α* protein expression in tumor cells, while suppressing miR-210 induced the high expression of GPDL1, which might become a new target in tumor treatment [20]. ACADL can encode an enzyme that participates in fatty acid and branched chain amino-acid metabolism. Hill et al. discovered that ACADL methylation might associate with the poor prognosis for breast cancer [21]. Regarding research on CPT2, Fujiwara et al. discovered that, in obesity- and nonalcoholic steatohepatitis-driven hepatocellular carcinoma, the downregulation of CPT2 accelerated tumor progression [22]. In clinical correlation analysis, the CPT2 expression quantities were significantly correlated with stage and pathological N stage, and its expression quantities gradually decreased as the stage and pathological N stage advanced, thus possibly meaning the reduced expression quantities of CPT2 in advanced CRC. Meanwhile, GO functional annotation suggested that GPD1L, AOC2, ACADL and CPT2, ACADL and AOC2, and ACADL were respectively active in oxidation-reduction process, fatty acid beta-oxidation, and electron carrier activity. Oxidation-reduction process was linked to the prognosis of hepatocellular carcinoma [23] and clear cell renal cell carcinoma [24]. The critical role of fatty acid beta-oxidation was also proven in the progression of cancer. It has been revealed that fatty acid beta-oxidation promotes proliferation of lymphatic endothelial cells by providing acetyl-CoA and regulates the differentiation of lymphatic endothelial cells with the epigenetic control of CPT1 [25]. And Wang et al. elucidated that JAK/STAT3-dependent fatty acid beta-oxidation is associated with breast cancer chemoresistance [26]. Xu et al. revealed that DNA methylation-driven genes in prostate adenocarcinoma were active in electron carrier activity [27]. What is more, KEGG pathway enrichment analysis uncovered that CPT2 and ACADL were both enriched in fatty acid degradation, fatty acid metabolism, and PPAR signaling pathway, which were all directly or indirectly involved in the process of lipid metabolism related to the progression of malignant tumors [28–31]. No doubt that the functional relationship among MRGs of the prognostic model provided compelling evidence for the role of metabolism in the progression of cancer from the molecular level. Upon the prognostic model constructed, Kaplan–Meier survival analysis for patients classified into high- and low-risk groups according to the median risk score in the training and validation groups verified the prognostic value of the prognostic model. The independent prognosis analysis validated that the risk score acquired had favorable statistical significance in predicting the patient prognostic outcomes. Additionally, the nomogram showed favorable accuracy in predicting the 1-, 3-, and 5-year survival rates of patients, contributing to systemically planning patient's follow-up.

We further explored the underlying multiple molecular relationships based on differentially expressed MRGs, among which, PPI network was constructed. From the PPI network, we identified 7 hub genes, namely, ATIC, IMPDH1, ENTPD8, AMPD2, GMPR, ENTPD3, and AMPD1. Ruan et al. found that the high expression quantity of IMPDH1 was related to the poor prognosis of malignant tumors, and the interaction between IMPDH1 and YB-1 was associated to the tumor metastasis, which might be a novel therapeutic target [32]. An et al. revealed that ENTPD8, the related gene of metabolite cytidine, was low expressed in pancreatic cancer [33]. And AMPD2 was identified as a potential biomarker for predicting the poor prognosis of undifferentiated pleomorphic sarcoma functional genomics identifies [34]. GMPR was found that it could downregulate GTP-bound Rho-GTPases and inhibited the further development of melanoma [35]. Feldbrugge et al. elucidated that the enzyme expressed by ENTPD3 prevented colon against inflammation and purinergic signaling regulated by ENTPD3 dominated neuroimmune interactions related to Crohn's disease [36]. AMPD1 could be regarded as the biomarker to predict the survival of breast cancer [37]. There is no doubt that our studies provided theoretical support for the interaction about MRGs in colorectal cancer. Besides, about the regulatory relationship of TFs on the prognosis-associated MRGs, we found that TFs (NR3C1, MYH11, MAF, and CBX7) positively regulated MRGs (ENPP2, PTGIS, GSTM5, and P4HA3). Previous study indicates that the point mutation of MYH11 and the reduced expression quantity of CBX7 are related to the poor prognosis for CRC [38, 39]. NR3C1 positively regulates the 4 prognosis-associated genes. Schlossmacher et al. indicated that glucocorticoid receptor encoded by NR3C1 promoted cell apoptosis through downregulating the expression of antiapoptotic proteins or inducing the expression of proapoptotic proteins [40]. However, there is no research on the regulatory role of NR3C1 in CRC, which may provide a new therapeutic target for metabolic treatment. Currently, targeted metabonomics analysis or nontargeted metabonomics analysis or the combination of both is employed to investigate the effect of differentially expressed metabolite or specific metabolite on the disease prognosis [41–43], among which, NMR spectroscopy is the representative of nontargeted metabonomics analysis method. Previously, Moolenaar et al. obtained the abnormally elevating N,N-dimethylglycine (DMG) induced by the congenital deficiency of enzyme dimethylglycine dehydrogenase (DMGDH) through 13C NMR spectroscopy and gas chromatography-mass spectrometry, which resulted in body odor [44]. In traditional metabonomics, the patient body fluid is collected to obtain the metabolic components to study the patient disease phenotype, but it is frequently dependent on the limited metabolic phenotype markers and is restricted by the quantities of sample metabolic components. Comparatively, our prognosis model utilized the high-throughput sequencing results to evaluate the DEGs and obtain their expression, and it was obtained based on the patient risk score acquired from the model algorithm, together with the patient clinical characteristics. Previously, the gene expression features are utilized to evaluate patient prognosis. O'Connell et al. constructed the multigene algorithms to quantify the prognosis for stage II/III CRC patients who received surgical treatment or combined with postoperative fluorouracil (FU) and leucovorin (LV) [45]. Agesen combined patients, populations, and Affymetrix exon-level microarrays to display a 13 gene-based classifier for predicting the prognosis of stage II CRC through COX regression analysis [46]. These studies have quantified the prognosis evaluation at the molecular level. Additionally, this MRG-based prognosis model expanded the gene biological functions. We conducted GSEA on high-risk group and low-risk group, finding numerous KEGG enriched pathways, most of which were related to metabolism. A vast majority of these pathways were enriched in the low-risk group, including propanoate metabolism pathway and fatty acid metabolism pathway. In recent years, research on the influence of lipid metabolic pathway on CRC development has always been a hotspot. Kazlauskas suggested that LPA played an important role in stimulating tumor angiogenesis [47], thus regulating tumor metastasis, while angiogenesis in tumor tissues usually promotes tumor growth and metastasis [48]. Yeh et al. employed the microarray-bioinformatics analysis methods to reveal that the activation of fatty acid pathway promoted CRC genesis and development at gene level [49]. Recently, Wang et al. discovered that the activation of the CPT1A-mediated fatty acid oxidation pathways suppressed anoikis to accelerate CRC development and metastasis [50]. And for propanoate metabolism pathway, Perroud et al. illustrated that 31 proteins in the propanoate metabolism pathway were associated with the genesis of clear cell RCC (ccRCC) [51]. However, how the propanoate metabolism pathway affects the genesis and development of colorectal cancer is still being probed. Moreover, compared with the high-risk group, it was feasible to apply the metabolic therapy in the low-risk CRC group. Such a result indirectly verified the feasibility to treat early CRC with metabolic-targeted therapy.

## 5. Conclusions

In conclusion, we constructed a prognostic model based on six MRGs to predict the prognosis of CRC by using the bioinformatics method. Univariate and multivariate Cox regression analysis for the training group, validation group, and overall samples verified the prognostic value of the prognostic model. Moreover, the TF-MRG network and PPI network revealed novel molecular regulatory targets about metabolism in CRC. GSEA for biological functions based on the prognostic model not only provided fresh sights about the therapeutic target but also facilitated the individualized treatment for CRC patients.

## Figures and Tables

**Table 1:** The grouping of patients.

Variables	Training (*n* = 306)	Validation (*n* = 128)
	Cases (percentage)	Cases (percentage)
Age		
≤60	90 (29.41%)	48 (37.50%)
>60	216 (70.59%)	80 (62.50%)
Gender		
Female	127 (41.50%)	68 (53.13%)
Male	179 (58.50%)	60 (46.88%)
Tumor stage		
Stage I	51 (16.67%)	26 (20.31%)
Stage II	118 (38.56%)	41 (32.03%)
Stage III	84 (27.45%)	35 (27.34%)
Stage IV	41 (13.40%)	23 (17.97%)
NA	12 (3.92%)	3 (2.34%)
T stage		
T1+Tis/T1	10 (3.27%)	3 (2.34%)
T2	53 (17.32%)	27 (21.09%)
T3	210 (68.63%)	87 (67.97%)
T4	33 (10.78%)	11 (8.59%)
N stage		
N0	178 (58.17%)	74 (57.81%)
N1	73 (23.86%)	34 (26.56%)
N2	44 (14.83%)	30 (23.44%)
Nx	1 (0.33%)	**\**
M stage		
M0	230 (75.16%)	96 (75.00%)
M1	40 (13.07%)	23 (17.97%)
Mx	31 (10.13%)	8 (6.25%)
NA	5 (1.63%)	1 (0.78%)

**Table 2:** The 12 prognosis-associated MRGs.

Genes	HR	HR.95L	HR.95H	*P* value
ACADL	2763.714	54.38402	140447.7	7.69*E*-05
ENPP2	1.071757	1.034298	1.110573	0.000135
GPX3	1.015988	1.006999	1.025058	0.000469
PTGIS	1.142533	1.057611	1.234274	0.000721
ADH1B	1.106607	1.043144	1.17393	0.000775
GPD1L	0.901048	0.839121	0.967546	0.004129
P4HA3	1.48786	1.133596	1.952835	0.004187
PAFAH2	0.847885	0.755554	0.951499	0.005029
CPT2	0.878993	0.80283	0.962382	0.005284
ADA	1.079064	1.021351	1.140038	0.006663
AOC2	2.620745	1.297612	5.293035	0.007223
GSTM5	3.900058	1.394631	10.90644	0.009489

**Table 3:** GO and KEGG functional enrichment analysis for the MRGs of the prognostic model.

Term	Count	Genes	*P* value
GO:0055114~oxidation-reduction process	3	GPD1L, AOC2, ACADL	0.011559
GO:0006635~fatty acid beta-oxidation	2	CPT2, ACADL	0.013035
GO:0009055~electron carrier activity	2	AOC2, ACADL	0.026378
hsa00071: fatty acid degradation	2	CPT2, ACADL	0.030166
hsa01212: fatty acid metabolism	2	CPT2, ACADL	0.034415
hsa03320: PPAR signaling pathway	2	CPT2, ACADL	0.047773

**Table 4:** Univariate independent prognosis analysis.

	Training group		Validation group		Overall samples	
	Hazard ratio	*P*	Hazard ratio	*P*	Hazard ratio	*P*
Age	1.021 (0.993-1.050)	0.147	1.064 (1.014-1.117)	0.011	1.030 (1.005-1.054)	0.016
Gender	0.828 (0.444-1.545)	0.554	2.008 (0.833-4.843)	0.121	1.071 (0.647-1.773)	0.789
Stage	3.100 (2.127-4.519)	<0.001	2.375 (1.442-3.911)	<0.001	2.857 (2.114-3.861)	<0.001
T	3.146 (1.715-5.771)	<0.001	3.781 (1.562-9.514)	0.003	3.337 (2.027-5.494)	<0.001
N	2.210 (1.549-3.154)	<0.001	2.662 (1.549-4.574)	<0.001	2.371 (1.764-3.186)	<0.001
M	7.199 (3.817-13.578)	<0.001	4.104 (1.740-9.680)	0.001	6.058 (3.640-10.080)	<0.001
Risk score	1.092 (1.059-1.126)	<0.001	1.161 (1.070-1.259)	<0.001	1.091 (1.063-1.119)	<0.001

**Table 5:** Multivariate independent prognosis analysis.

	Training group		Validation group		Overall samples	
	Hazard ratio	*P*	Hazard ratio	*P*	Hazard ratio	*P*
Age	1.037 (1.008-1.068)	0.013	1.094 (1.037-1.155)	<0.001	1.039 (1.014-1.064)	0.002
Gender	0.625 (0.321-1.218)	0.167	2.089 (0.814-5.356)	0.125	0.904 (0.537-1.522)	0.704
Stage	2.418 (0.767-7.621)	0.132	1.793 (0.351-9.417)	0.482	2.042 (0.833-5.008)	0.119
T	1.532 (0.789-2.978)	0.208	2.171 (0.740-6.639)	0.158	1.744 (0.986-3.084)	0.056
N	1.098 (0.573-2.104)	0.777	1.731 (0.640-4.682)	0.280	1.215 (0.735-2.009)	0.447
M	1.445 (0.287-7.288)	0.655	0.773 (0.091-6.537)	0.813	1.308 (0.384-4.459)	0.667
Risk score	1.056 (1.021-1.092)	0.002	1.178 (1.071-1.295)	<0.001	1.059 (1.030-1.089)	<0.001

**Table 6:** The ten representative KEGG pathways in high- and low-risk groups.

Names	Size	ES	NES	NOM *P*-value	FDR *q*-value
High-risk group					
KEGG_complement_and_coagulation_cascades	69	0.684	2.100	0.002	0.030
KEGG_basal_cell_carcinoma	55	0.620	2.077	0.002	0.022
KEGG_glycosaminoglycan_biosynthesis_chondroitin_sulfate	22	0.795	2.048	0	0.021
KEGG_ECM_receptor_interaction	84	0.708	2.021	0.006	0.022
KEGG_autoimmune_thyroid_disease	50	0.724	2.018	0.002	0.018
Low-risk group					
KEGG_propanoate_metabolism	32	−0.842	−2.357	0	0
KEGG_peroxisome	78	−0.725	−2.328	0	0
KEGG_fatty_acid_metabolism	42	−0.776	−2.302	0	4.22*E*-04
KEGG_valine_leucine_and_isoleucine_degradation	43	−0.816	−2.249	0	3.71*E*-04
KEGG_butanoate_metabolism	34	−0.764	−2.147	0	0.003

## Data Availability

We analyzed the publicly available datasets in this study. All data were extracted from TCGA, MSigDB, and Cistrome Project.

## Supplementary Materials

Supplementary materials Table S1: the 6475 DEGs. Table S2: the 289 differentially expressed MRGs. Table S3: the 72 differentially expressed TFs.
